# *Bryorutstroemia* (*Rutstroemiaceae*, *Helotiales*), a New Genus to Accommodate the Neglected Sclerotiniaceous Bryoparasitic Discomycete *Helotium fulvum*

**DOI:** 10.3390/life13041041

**Published:** 2023-04-18

**Authors:** Hans-Otto Baral, Zuzana Sochorová, Michal Sochor

**Affiliations:** 1 Independent Researcher, Blaihofstr. 42, D-72074 Tübingen, Germany; 2 Department of Botany, Faculty of Science, Palacký University Olomouc, Šlechtitelů 27, CZ-78371 Olomouc, Czech Republic; 3 Centre of the Region Haná for Biotechnological and Agricultural Research, Crop Research Institute, Šlechtitelů 29, CZ-78371 Olomouc, Czech Republic

**Keywords:** *Bucklandiella*, *Clarireedia*, *Dicranella*, *Dicranum*, elongation factor-1alpha (*EF1α*), fungal diversity, nuITS+LSU rDNA, sandstone, vital taxonomy

## Abstract

The new genus *Bryorutstroemia* is established for the red-brown, stipitate, bryoparasitic discomycete *Helotium fulvum* Boud. Combined phylogenetic analysis of ITS and LSU rDNA and *EF1α* revealed that *Bryorutstroemia fulva* belongs to the sclerotiniaceous clade, which comprises the paraphyletic families *Rutstroemiaceae* and *Sclerotiniaceae. Bryorutstroemia* formed with *Clarireedia* a supported clade (*Rutstroemiaceae* s.l.), though with high distance. *Bryorutstroemia* closely resembles other *Rutstroemiaceae* in having uninucleate ascospores with high lipid content and an ectal excipulum of textura porrecta, but is unique because of its bryophilous lifestyle and is extraordinary with its thick-walled inamyloid ascus apex. Although *B*. *fulva* was described in 1897, very few records came to our notice. The present study summarizes the known distribution of the species, including 25 personal collections from the years 2001–2022. *Bryorutstroemia fulva* was most often encountered on *Dicranella heteromalla,* and rarely on other members of *Dicranales* or *Grimmiales*, while inducing necrobiosis of the leaves. A detailed description based on mainly fresh apothecia is provided together with a rich photographic documentation. Six new combinations are proposed based on our phylogenetic results and unpublished personal morphological studies: *Clarireedia asphodeli*, *C. calopus*, *C. gladioli*, *C. henningsiana*, *C. maritima*, and *C. narcissi*.

## 1. Introduction

*Helotium fulvum* was described by Boudier in 1897 based on his collection from Forêt de Carnelle north of Paris (France) [1]. The apothecia grew on sandy soil among *Phascum*, *Dicranella*, and other small mosses. They consistently arose from leaf axils (leaf bases) at the tip of stems of what was obviously a *Dicranella*. Reliable reports of the species in the literature are very sparse up to now and include collections from Great Britain [2] and Belgium [3]. During an ascomycete foray in Luxembourg in April 2001 [4], the first author collected and documented a single apothecium on *Dicranella*, which remained undetermined for many years. Because of its very short stipe, large, ellipsoid, multiguttulate ascospores, and large, inamyloid asci, an affinity with the genus *Mniaecia* Boud. was considered, despite its reddish-brown colour. Further fresh collections from Sweden (Småland), France (Bretagne), Germany (Sachsen), Czech Republic (regions of Ústí nad Labem, Liberec, Hradec Králové, Vysočina, Olomouc, Moravian-Silesian and Zlín), Poland (Silesia), and Hungary (near Budapest) all deviated from our first record in possessing comparatively long stipes. Apart from the brown, stipitate apothecia, a ± gelatinized ectal excipulum of textura (prismatica-)porrecta with ochre-brown, partially encrusted cortical cells pointed to a relationship with the genus *Rutstroemia* P. Karst. The aim of our work was to clarify the phylogenetic position of *Helotium fulvum*, summarize its known distribution, and provide a detailed description based on recent collections.

## 2. Materials and Methods

### 2.1. Sampling and Observation

Macro- and microscopic characters were studied from fresh apothecia, predominantly from living (*) elements following the standards of vital taxonomy [5], in comparison also with samples from dead (†) elements. Apothecia were rehydrated after different intervals for testing their drought tolerance. Tap water (H_2_O) was used as a mounting medium. Colour reactions were tested with IKI and KOH. The latter was also applied for testing pigment solubility, resistance of oil drops (LBs), and iodine reactions under KOH-pre-treatment. CX21 (Olympus, Czech Group, Prague, Czech Republic) and Zeiss Standard 14 microscopes, with magnifications of 40×, 100×, 400×, and 1000×, were used in our study. Measurements were conducted in tap water, either directly or on photographs using the Piximètre 5.10 software [6] or by calculating from scales prepared using a Zeiss calibration slide.

Collections are deposited in the herbaria of PRA (Z. Palice), PRM (Z. Sochorová), and UPS (R. Isaksson), and in the private herbaria of H.O. Baral (H.B.), M. Lüderitz (M.L.), C. Németh (C.N.), and J.P. Priou (J.P.P.).

The following abbreviations are used: H_2_O = tap water, KOH = potassium hydroxide (~5%), LBs = lipid bodies (oil drops), VBs = refractive KOH-soluble vacuolar bodies, IKI = ~1% iodine (I_2_) in 3% KI (potassium iodide), MLZ = Melzer’s reagent, OCI = lipid content, PVA = polyvinyl acetate, idem = the same, ibid. = from the same geographical region, l.c. = reference cited, doc. vid. = documentation seen, non vid. = no documentation seen. Values in {} indicate the number of collections, thereby numbers after a slash refer to uncertain hosts.

### 2.2. DNA Extraction, PCR Amplification and Sequencing

DNA was extracted from dried apothecia using the CTAB method described by Doyle et Doyle [7]. Apothecia were homogenised using a pestle and incubated in 300 µL of extraction buffer at 65 °C for one hour. The extract was subsequently purified in chloroform-isoamyl alcohol mixture (24:1), precipitated by isopropanol, washed in 70% ethanol, dried and finally dissolved in water and incubated with RNase for 30 min at 37 °C. DNA quality was checked using agarose gel electrophoresis. Three genomic regions including the internal transcribed spacers (ITS = ITS1-5.8S-ITS2 region) and the 28S subunit (LSU) of ribosomal DNA (rDNA) plus the translation elongation factor-1alpha (*EF1α*) were amplified and sequenced with the primers ITS1F [8] / ITS4 [9], LR0R/LR6 [10], and EF1-983F/EF1-1567R [11], respectively. PCR was performed with EliZyme FAST Taq MIX Red (Elisabeth Pharmacon, Brno, Czech Republic) following a standard protocol with 37 cycles and annealing temperature of 54 °C. The PCR products were purified by precipitation with polyethylene glycol (10% PEG 6000 and 1.25 M NaCl in the precipitation mixture) and sequenced from both directions using the same primer pairs by the Sanger method at Macrogen Europe, Amsterdam, the Netherlands.

### 2.3. Phylogenetic Analysis

Specimens used in the phylogenetic analysis are listed in Table 1. Newly generated sequences were edited using the Geneious software (ver. 7.1.7., Biomatters, Auckland, New Zealand). Alignment was achieved with MAFFT plugin and subsequently manually checked. Phylogeny was reconstructed using the Maximum Likelihood (ML) method with the substitution model GTR+G+I tested by bootstrapping, using 1000 pseudoreplicates in MEGA (ver. 6.06) [12]. Bayesian phylogeny inference (BI) was computed in MrBayes (ver. 3.2.4) [13] using the GTR+I+G (for ITS), SYM+I+G (LSU) and GTR+G (*EF1α*) substitution model, as determined by AICc in PartitionFinder 2.1.1 [14]. Besides the combined trees, single gene trees were calculated. The analysis was run for 15 million generations in four independent runs, sampling every 1000th generation and excluding the first 50% of generations as burn-in, and temperature parameter was set to 0.05 for better chain mixing. The Basic Local Alignment Search Tool (BLAST) [15] was used for searching similar sequences in publicly available sequence databases [16].

Maximum Likelihood (ML) phylogenetic analysis was performed in MEGA6 with the settings ‘use all sites, nearest-neighbour-interchange, weak branch swap filter’. Distance analyses were performed with MEGA6 using the settings ‘p-distances, transitions + transversions, uniform rates, pairwise deletion’.

## 3. Results

### 3.1. Taxonomy

***Bryorutstroemia*** Sochorová and Baral, gen. nov.—MycoBank MB 847031

Diagnosis: Differs from *Rutstroemia* and *Clarireedia* by its inamyloid asci, bryoparasitic habitat, and genetic profile.

Etymology: named after the bryicolous habitat and the similarity with the genus *Rutstroemia*.

Type: *Bryorutstroemia fulva* (Boud.) Sochorová, Baral and Priou

***Bryorutstroemia fulva*** (Boud.) Sochorová, Baral and Priou, comb. nov.—MycoBank MB 847033

Figure 1, Figure 2, Figure 3, Figure 4, Figure 5, Figure 6, Figure 7 and Figure 8

Basionym: *Helotium fulvum* Boud., Bull. Soc. mycol. Fr. 13(1): 16 (1897)

≡ *Hymenoscyphus fulvus* (Boud.) Hengstm., in Arnolds et al., Overzicht paddest. Nederl.: 654 (1985)

Etymology: after the red-brown apothecial colour caused by brown wall deposits on paraphyses and cortical hyphae of ectal excipulum.

Holotype: France, Île-de-France, Val d’Oise, Paris, Forêt de Carnelle, on *Dicranella* cf. *heteromalla*, II.1896, É. Boudier (doc. vid.).

**Apothecia** (0.4–)0.5–1(–1.5) mm diam. when fresh {16}, receptacle 0.25–0.33 mm thick at lower flanks, 0.2–0.26 mm thick at margin {3}, singly or rarely in fascicles of two to four fused at the base, non-gelatinous; disc rounded in upper view, flat, eventually slightly convex, light to mostly bright to deep reddish- to purplish-brown (carmine-brown), also ochre-brown to dark brown, non-translucent, margin distinct, not protruding, even, exterior concolorous, flesh pale brown; mostly with a distinct **stipe** (0.1–)0.5–1.5(–1.8) × (0.12–)0.15–0.3(–0.55) mm {12}, cylindrical or widened above or sometimes below, pale to deep red-brown, basal (1/10–)1/4–1/3 of stipe blackish-brown {15}, base inserted in leaf axils at tip of stem, seemingly superficial. **Asci** *(150–)170–220(–233) × (17–)18–24(–27) µm {8}, †(100–)110–155(–165) × (12–)13–17(–18) µm {4}, cylindric-clavate, eight-spored, spores *obliquely biseriate, pars sporifera *(50–)60–70(–87) µm long if all eight ascospores fully developed, living mature asci protruding 20–50 µm beyond paraphyses; **apex** */†obtuse or slightly to strongly conical, dome immature †(4–)5–7(–10) µm thick (*2–2.5 µm), mature †3–7 µm (*1–1.2 µm) {9}, IKI– {22}, MLZ– {5}, when KOH-pretreated IKI–/MLZ– {1}, dome hemispherically protruding into ascoplasm, without apical chamber, lateral ascus wall †0.5–1 µm thick, subapically †1.2–1.5 µm; **base** with medium to long stalk, arising from simple septa {21} with basal downward-oriented protuberance {11}, sometimes bifurcate by one branch forming the protuberance {4}. **Ascospores** *(14–)16–25(–27.5) × (6–)7–10(–11) µm {13} [*Q = 2.27–2.76–4.2 (n = 50), *Me = 23.1 × 8.4 µm, Z.S. 2/2021; *Q = 2.16–2.57 (n = 20), C.N. 103], †(14.5–)16–22(–24.7) × (5.5–)6–8(–9) µm {3} [†Q = 2.1–2.6–3(–3.8) (n = 50), †Me = 18.6 × 7.2 µm, Z.S. 2/2021], ellipsoid, also cylindric-ellipsoid or ellipsoid- to fusoid-clavate, homopolar, straight, ends obtuse, smooth; containing numerous **LBs** of (0.5–)0.8–2(–2.5) µm diam. (multiguttulate) {22}, LBs in young spores much smaller and more numerous, OCI 4.5–5 {20}, leaving an area occupied by the single nucleus, when freshly ejected sometimes surrounded by a sheath that separates from the spore wall {7} (Figure 7: 1e,3,6); overmature spores one-septate {12}, hyaline, rarely germinating with one hypha at the pole or more laterally. **Paraphyses** cylindrical-filiform throughout, sometimes slightly clavate above, rarely slightly capitate, spathulate, or narrowly obtusely-sublanceolate, straight to slightly flexuous, sometimes curved under a wide arc, hyaline, terminal cell *21–53 {4} × (1.7–)2–3(–3.4) µm {6}, †(14–)19–45(–50) {3} × (1.5–)2–2.5(–3) µm {4}, without VBs {15}, sometimes with groups of LBs {1}, embedded above in (very) pale fox-brownish, smooth, gel-like exudate, lower cells *16–30 × 1.7–3.1 µm {2}, †1.8–2 µm wide {1}; sparsely to frequently branched in middle part. **Subhymenium** hyaline, *17–33 µm thick, non-gelatinized, cells angular, subglobose or irregular, *5–11 × 3–8 µm. **Medullary excipulum** with pale brass-ochre to brownish intercellular exudate, non-gelatinized, *90–150 µm thick in centre, *60–120 µm at lower flanks, in receptacle of dense textura intricata with tendency to an upward orientation, cells *8–24 × 2.5–5.5(–11) µm {2}, thin-walled, sharply delimited from ectal excipulum by a thin, parallel, pale brown layer of t. porrecta; in stipe of vertically oriented hyaline to pale brown t. porrecta, cells cylindrical, *(13–)20–60(–75) × (3.5–)5–6(–11) µm {2}. **Ectal excipulum in receptacle** of hyaline to pale ochre-brown, thin-walled, *not or slightly (†medium to strongly) gelatinized, textura (prismatica-)porrecta from base to margin, oriented at a (0–)10–30(–50)° angle to the surface (often very irregularly, Figure 5: 3b), *(30–)40–55 µm thick at lower flanks, cells *(10–)15–33(–48) × 3.5–7(–9) µm {2}, †19–28 × 3.5–6 µm {1}; *20–40 µm thick near margin, smaller-celled, bright reddish-brown, marginal cortical cells *12–16 × 3–3.5 µm {1}, ± flexuous, forming hair-like elements; cortical cells of similar size, with pale to bright ochre- to red-brown, thin, smooth {2} or granular to ridge-like encrustation {6} (Figure 6: 1a,2a), in surface view straight to sometimes ± undulating, often with short, scattered lateral protrusions, *5–14 × 3.5–5 µm {3}; **in stipe** of not to slightly gelified t. porrecta oriented parallel to the surface, formed by cylindrical, often anastomosing or branching, thin- to thick-walled (*0.2–0.7 µm) cells *12–40 × 2.7–7(–9.5) µm {1}; cortical cells as on receptacle. **Tissues** without crystals, without IKI reaction, excipular pigment in KOH not changing colour, not dissolved {3}. **Anchoring hyphae** sparse, brown, forming chains of †8–12 × 5–6.5 µm large cells, walls †~0.5–0.8 µm thick {1}. **Anamorph** unknown.

**Habitat**: on leaf axils of living or mainly dead individuals of *Bucklandiella heterosticha* {1}, *Dicranella cerviculata* {1}, *D. heteromalla* {35/2}, *Dicranella* sp. {1}, *Dicranum scoparium* {2}, causing yellowish discolouration of the host, mosses growing on rocks or equally often on sandy to loamy or humous soil. **Associated** (± remotely): *Mniaecia* cf. *gemmata* {3}, *M. jungermanniae* {4}. **Drought tolerance**: only a few ascospores survived when dry apothecia were examined after 10 days up to 2 ⅓ months. **Altitude**: 10–530(–835) m above sea level. **Phenology**: X–VII(VIII–IX) (throughout the year, especially in winter and spring). **Geology**: Bretagne: acidic quartzite, sandstone, argillaceous siltite, shale-like schist (Ordovician, Brioverian); Luxembourg: Lower Lias (sandstone); Czechia and Poland: acidic sandstone, alluvial sediments, gneiss, migmatite, granulite.

**Specimens included**: **Sweden**: **Småland**, **Jönköpings län**, 4 km WNW of Sävsjö, 3.5 km SSW of Bringetofta, 0.5 km SSE of Rickelstorp, 245 m, *Bucklandiella heterosticha* on silicate stonewall, 13.XII.2020, R. Isaksson (UPS F-990878).—1.3 km SSE of Rickelstorp, 235 m, on *Dicranum scoparium* on silicate stonewall, 29.XII.2020, R. Isaksson (doc. vid.).—**Great Britain**: **Scotland**, **East Lothian**, SSE of Haddington, Gifford, ~120 m, on *Dicranella heteromalla*, 18.X.1964, D.M. Henderson (E, non vid.).—idem, 25.X.1965.—idem, X.1968.—idem, 10.X.1969.—**Southwest England**, **West Gloucestershire**, 30 km N of Bristol, Rodmore Grove, 140 m, host not stated, 1.IX.1991, A. Yelland (non vid.) [17].—**Netherlands**: **Groningen**, 2.5 km S of Vlagtwedde, 1 km NW of Weende, Liefstinghsbroek, 10 m, on *D. heteromalla*, 2.II.2022, J. Boers (unpreserved, doc. vid.).—**Belgium**: **Vlaanderen**, **Antwerpen**, 11.5 km NE of Antwerpen, 4 km NE of Schoten, La Garenne, 12 m, on *D. cerviculata*, 24.II.1992, J. Slembrouck and H. De Meulder (H.B. 4632).—**France**: **Bretagne**, **Côtes-d’Armor**, 4.5 km WNW of Mur-de-Bretagne/Guerléda, 1 km SW of Caurel, Lac de Guerlédan, 133 m, on *D. heteromalla*, 7.III.2005, J.P. Priou (J.P.P. 15051).—**Ille-et-Vilaine**, 4.5 km E of La Gacilly, 1.2 km SE of Sixt-sur-Aff, Dessous Le Guerche, D255, 66 m, on *D. heteromalla*, 3.III.2006, J.P. Priou (J.P.P. 26054, H.B. 8083).—**Morbihan**, 3 km SW of La Gacilly, 2.8 km NW of Glénac, route de La Forêt Neuve, 80 m, on *D. heteromalla*, 6.IV.2004, J.P. Priou (J.P.P. 24120).—3.7 km S of Montfort-sur-Meu, 2 km WSW of Talensac, 110 m, on *D. heteromalla*, 19.III.2021, J.P. Priou (J.P.P. 2021050, non vid.)—**Île-de-France**, **Val d’Oise**, ~29 km N of Paris, Forêt de Carnelle, ~200 m, on *D.* cf. *heteromalla*, II.1896, É. Boudier (holotype, doc. vid.).—**Luxembourg**: **Gutland**, Petite Suisse, 11.5 km WNW of Echternach, 2.2 km W of Beaufort, Esselbur, Elteschmuer S of Tinnes, 405 m, on *D.* cf. *heteromalla*, 25.IV.2001, H.O. Baral (H.B. 6917 [PVA-slide]).—**Germany**: **Niedersachsen**, 5 km ESE of Ratzeburg, ~2.8 km WSW of Mustin, SW of Garrensee, NW of Garrenseeholz, on *D. heteromalla*, 9.III.1995, M. Lüderitz (M.L., non vid.) [18].—**Sachsen**, 8.5 km SSW of Zittau, 0.8 km S of Kurort Oybin, 465 m, on *D. heteromalla* on a sandstone rock, 15.V.2021, Z. Sochorová (ex Z.S. 44/2021, PRM 956027).—**Bayern**, **Oberbayern**, near Ingolstadt, ~400 m, on *D. heteromalla*, 31.VIII.1979, J. Poelt (Plantae Graecensis 255, PDD 60714, non vid.)— **Poland: Lower Silesian Voivodeship**, 16 km SE of Wałbrzych, 1.6 km SE of Walim, Owl Mountains landscape park, 775 m, on *D. heteromalla* on soil, 17.IV.2022, Z. Sochorová (ex Z.S. 1/2022, PRM 957650).—**Czech Republic**: **Ústí nad Labem region**, **Děčín district**, 5 km N of Jetřichovice, České Švýcarsko National Park, Křinice valley, ENE of Jankův kopec, 348 m, on *D. heteromalla* on soil, 10.XI. 2021, Z. Palice, I. Marková and P. Uhlík (ex Z.P. 32329, PRA, vid.).—**Liberec region, Česká Lípa district**, 6.5 km NNE of Česká Lípa, 1.3 km NW of Svojkov, 1 km SSW of Sloup v Čechách, group of rocks above the road no. 268, 350 m, on *D. heteromalla* on a sandstone rock, 1.I.2021, Z. Sochorová (ex Z.S. 2/2021, PRM 956016, sq.: ITS OP035812).—idem, 28.II.2021 (ex Z.S. 11/2021, PRM 956020).—10 km ENE Mimoň, 2.8 km SE of Hamr na Jezeře, Divadlo Nature Monument, 375 m, on *D. heteromalla* on a sandstone rock, 27.II.2021, Z. Sochorová (ex Z.S. 8/2021, PRM 956018).—3.2 km S of Hamr na Jezeře, 2.7 km NE Svébořice, Stohánek Nature Monument, 350 m, on *D. heteromalla* on a sandstone rock, 27.II.2021, Z. Sochorová (ex Z.S. 9/2021, PRM 956019, sq.: ITS + LSU OP035829, *EF1α* OP058104).—**Liberec district**, 21 km WNW of Liberec, 4 km N of Jablonné v Podještědí, 1.2 km SW of Petrovice, 410 m, on *D. heteromalla* on soil, 4.III.2021, Z. Sochorová (ex Z.S. 17/2021, PRM 956023).—2.1 km NE of Jablonné v Podještědí, 345 m, on *D. heteromalla*, 4.VII.2021, Z. Sochorová (ex Z.S. 61/2021, PRM 956032).—idem, 15.XI.2021 (ex Z.S. 153/2021, PRM 956457).—1.8 km NE of Jablonné v Podještědí, at St. Zdislava’s spring, 335 m, on *D. heteromalla*, 4.VII.2021, Z. Sochorová (ex Z.S. 62/2021, PRM 956033).—2 km ENE of Jablonné v Podještědí, 1 km S Lvová, 365 m, on *D. heteromalla* on a sandstone rock, 1.III.2021, Z. Sochorová (ex Z.S. 15/2021, PRM 956021).—4.5 km E of Jablonné v Podještědí, 0.5 km N of Janovice v Podještědí, 260 m NNW of cemetery, 370 m, on *D. heteromalla* on a sandstone rock, 3.III.2021, Z. Sochorová (ex Z.S. 16/2021, PRM 956022).—9 km SW of Liberec, 0.8 km SE of Rozstání pod Ještědem, Horka forest park, 460 m, on *D. heteromalla*, 3.VII.2021, Z. Sochorová (ex Z.S. 60/2021, PRM 956031).—3 km SW of Česká Lípa, Peklo National Nature Monument, 270 m, on *D. heteromalla*, 16.XI.2021, Z. Sochorová (ex Z.S. 158/2021, PRM 956458).—**Jablonec nad Nisou district**, 2.3 km WNW of Koberovy, 1 km NW of Besedice, 445 m, on *D. heteromalla* on soil, on sandstone bedrock, 26.II.2021, Z. Sochorová (ex Z.S. 7/2021, PRM 956017, sq.: ITS + LSU OP035830, *EF1α* OP058105).—**Hradec Králové region, Náchod district**, Broumovské stěny National Nature Reserve, 6 km SSW of Broumov, 1.6 km ENE of Slavný, 650 m, on *D. heteromalla* on soil on sandstone bedrock, 18.IV.2022, Z. Sochorová (ex Z.S. 2/2022, PRM 957651).—idem, 0.9 km ENE of Slavný, Zaječí rokle, 605 m (ex Z.S. 4/2022, PRM 957652).—**Vysočina region**, **Havlíčkův Brod district**, Údolí Doubravy Nature Reserve, 3.5 km ESE of Chotěboř, 820 m WNW of Bílek railway station, 545 m, on *D. heteromalla* on soil over migmatite to orthogneiss, 21.V.2021, Z. Sochorová (ex Z.S. 48/2021, PRM 956029).—ibid., 600 m WNW of Bílek railway station, 545 m, on *D. heteromalla* on soil, 21.V.2021, Z. Sochorová (ex Z.S. 47/2021, PRM 956028).—**Olomouc region**, **Olomouc district**, Dolany u Olomouce, W of Nové Sady, 305 m, on *D. heteromalla* on soil, 28.III.2021, Z. Sochorová (ex Z.S. 18/2021, PRM 956024).—ibid., S of Nové Sady, 340 m, on *D. heteromalla* on soil-stony bedrock, 28.III.2021, Z. Sochorová (ex Z.S. 19/2021, PRM 956025, sq.: ITS + LSU OP035828, *EF1α* OP058103).—**Šumperk district**, 4.6 km NW of Staré Město, 1 km NNW of the church in Stříbrnice, 835 m, on *D. heteromalla* on soil, 30.V.2021, Z. Sochorová (ex Z.S. 58/2021, PRM 956030).—**Moravian-Silesian region**, **Opava district**, 2 km NW of Těškovice, 360 m, on *D. heteromalla* on soil, 4.IV.2021, Z. Sochorová (ex Z.S. 23/2021, PRM 956026).—**Zlín region**, **Zlín district**, 5 km SE of Bystřice, 2 km SSE of Hostýn, 690 m, on *D. heteromalla* on soil, 28.X.2022, Z. Sochorová (ex Z.S. 136/2022, PRM 958329).—**Hungary**: **Pest county**, **Budakeszi district**, 12 km WNW of Budapest, 3 km W of Budakeszi, 320 m, on *Dicranum scoparium* on soil, 8.XII.2020, C. Németh (C.N. 103, sq.: ITS + LSU OP035831, *EF1α* OP058106).

### 3.2. Phylogeny

ITS sequences were obtained from five collections of *B. fulva*, while LSU and *EF1α* were obtained from four (Table 1). The S1506-intron is absent in all of them, according to the used ITS1F primer. The five sequences are fully identical in the overlapping parts. In BLASTn searches in GenBank, *B. fulva* had the highest ITS similarity to members of the *Clarireedia* clade: *Clarireedia narcissi* (90%), *C. monteithiana* and *C. jacksonii* (89.5%), *C. asphodeli*, *C. calopus*, *C. henningsiana*, *C. homoeocarpa*, *C. maritima*, and *C. paspali* (88–89%). Also in the LSU D1–D2 domain the highest similarity (95%) was to members of *Clarireedia* but also to *Piceomphale*, followed by other rutstroemiaceous taxa, including *Rutstroemia firma* (92.5–93.5%).

*Helotium fulvum* was only once recombined into another genus when Hengstmengel [19] suggested a relationship with the genus *Hymenoscyphus.* Our phylogenetic analysis of nuITS+LSU rDNA + *EF1α* (Figure 9 and Figure S4), in which we used *Hymenoscyphus scutula* (Pers.) W. Phillips (isolate G.M. 2014-12-25.2, ITS + LSU: MK674606) as outgroup, indicated a high distance between *B. fulva* and that species. Instead, *B. fulva* nested in the strongly supported sclerotiniaceous lineage as circumscribed by Baral [20] p. 173, a group which currently includes two families, *Rutstroemiaceae* and *Sclerotiniaceae*. Two further families in our dataset, *Cenangiaceae* and *Chlorociboriaceae*, clustered outside the sclerotiniaceous lineage.

In our Bayesian analysis, the paraphyletic family *Rutstroemiaceae* appears in three different clades (Figure 9 and Figure 10). One clade (*Rutstroemiaceae* s.str.) comprises species growing on wood and bark but also on the leaves of trees; it includes two strongly supported subclades, one containing the type species of *Rutstroemia*, *R. firma*, and four other *Rutstroemia* spp., but also *Torrendiella setulata,* the other containing *Lambertella subrenispora* and *Lanzia allantospora*.

A different, strongly supported clade comprises species growing on monocots and also on dung. It represents the recently established genus *Clarireedia* L.A. Beirn et al. [21], with the type species *C. homoeocarpa* (F.T. Benn.) L.A. Beirn et al. (≡ *Sclerotinia homoeocarpa* F.T. Benn.), and includes species currently assigned to *Rutstroemia* but also *Ciboria*, *Sclerotinia*, and *Stromatinia*. Within *Clarireedia*, *C. paspali* clustered in our ITS+LSU analysis closest to *B. fulva* despite its comparatively high ITS distance (Figure 10), perhaps because the specimen lacks LSU, whereas in our ITS+LSU+*EF1α* analysis it clustered supported with other *Clarireedia* spp. (Figure 9).

The following new combinations are proposed to harmonize the nomenclature of species on monocots which cluster in the supported *Clarireedia* clade. The listed taxonomic synonyms are to be taken as tentative and require type studies for clarification. *C. henningsiana* (= *R. paludosa*) is here understood as a species on *Cyperaceae* and *Juncaceae* characterized by simple-septate asci, whereas *C. calopus* (= *C. bennettii*) and *C. maritima* represent species on *Poaceae* characterized by asci arising from croziers, *C. maritima* also by asci with inamyloid, moderately thick-walled apex (pers. obs.). We tentatively regarded *R. cuniculi* as a synonym of *C. calopus* because available ITS sequences in GenBank differed from those of *C. calopus* by only one nucleotide.

***Clarireedia asphodeli*** (Duvernoy and Maire) Baral and Sochorová, comb. nov.—MycoBank MB 847034

Basionym: *Ciboria asphodeli* Duvernoy and Maire, in Maire, Bull. trimest. Soc. mycol. Fr. 44: 54 (1928)

*≡ Rutstroemia asphodeli* (Duvernoy and Maire) R. Galán and Matočec, in Galán et al., Mycologia 107(4): 799 (2015)

***Clarireedia gladioli*** (Drayton) Baral and Sochorová, comb. nov.—MycoBank MB 847035

Basionym: *Sclerotinia gladioli* Drayton, Phytopathology 24: 397 (1934)

*≡ Stromatinia gladioli* (Drayton) Whetzel, Mycologia 37(6): 674 (1945)

***Clarireedia henningsiana*** (Plöttn.) Baral and Sochorová, comb. nov.—MycoBank MB 847036

Basionym: *Ciboria henningsiana* Plöttn., in Maire, Verh. bot. Ver. Prov. Brandenb. 41: X (1899)

= *Rutstroemia paludosa* (E.K. Cash and R.W. Davidson) J.W. Groves and M.E. Elliott, Can. J. Bot. 39: 225 (1961)

= *Ciboria blanda* Svrček, Česká Mykol. 12(4): 225 (1958)

***Clarireedia maritima*** (Roberge ex Desm.) Baral and Sochorová, comb. nov.—MycoBank MB 847037

Basionym: *Peziza maritima* Roberge ex Desm., Ann. Sci. Nat., Bot., sér. 3 3: 366 (1845)

≡ *Rutstroemia maritima* (Roberge ex Desm.) Dennis, Persoonia 3(1): 52 (1964)

***Clarireedia narcissi*** (Drayton and J.W. Groves) Baral and Sochorová, comb. nov.—MycoBank MB 847038

Basionym: *Stromatinia narcissi* Drayton and J.W. Groves, Mycologia 44(1): 126 (1952)

***Clarireedia calopus*** (Fr.) Baral and Sochorová, comb. nov.—MycoBank MB 847039

Basionym: *Peziza calopus* Fr., Observ. mycol. (Havniae) 2: 307 (1818)

≡ *Rutstroemia calopus* (Fr.) Rehm, Rabenh. Krypt.-Fl., Edn 2 (Leipzig) 1.3(lief. 39): 768 (1893) [1896]

= *Clarireedia bennettii* C. Salgado, L.A. Beirn, B.B. Clarke and J.A. Crouch, in Salgado-Salazar et al., Fungal Biology 122(8): 769 (2018)

= *Rutstroemia cuniculi* (Boud.) M.E. Elliott, Can. J. Bot. 45(4): 521 (1967)

A third clade is formed by the type species of *Lambertella*, *L. corni-maris*, and two more *Lambertella* spp., but also includes *Bicornispora seditiosa*, and with less support *Rutstroemia longipes* and *Martininia panamaensis*. A further, strongly supported clade represents the family *Sclerotiniaceae*, which includes in the present analysis members of *Ciboria*, *Dumontinia*, *Monilinia*, *Pycnopeziza*, *Schroeteria*, *Sclerencoelia*, and *Sclerotinia*, with partly high distances among the species.

Four species of the sclerotiniaceous lineage clustered outside the four above-mentioned clades (Figure 9 and Figure 10): *Bryorutstroemia fulva* formed with *Clarireedia* a strongly supported clade, though with high distance. *Scleromitrula shiraiana* is morphologically similar to *Ciboria* but it clustered unsupported in Figure 9 but formed a moderately supported sister clade to *Rutstroemia* s.str. in Figure 10. As in other published analyses [22], *Piceomphale bulgarioiodes* clustered with “*Cenangium*” *acuum* distant from all other sclerotiniaceous taxa, despite its morphological similarity with *Ciboria* and an ascus structure of the *Sclerotinia*-type. The two species form the “*Piceomphale*-clade”, which is difficult to assign to a family, but may be better recognized in the sclerotiniaceous lineage than in *Cenangiaceae* to which *Encoelia furfuracea* belongs [22].

A phylogenetic tree generated with MEGA6 (ML, GTR+G+I, 1000 replicates, Figure S4), based on the very same dataset as in Figure 9, gave a similar tree topology though with only weak support for *Rutstroemia* s.str. and moderate support for *Lambertella* s.str. Again, *B. fulva* clustered sister to *Clarireedia*, though with only moderate support and by forming with *C. paspali* an unsupported clade. Contrary to the Bayesian analysis, *Martininia panamaensis* clustered strongly supported with *Lambertella* in the ML ITS+LSU analysis of Baral et al. [23] but unresolved in Figure S4, and *Scleromitrula shiraiana* clustered unresolved in both Baral et al. [23] and in Figure S4.

## 4. Discussion

### 4.1. Morphological Remarks

*Bryorutstroemia fulva* is characterized by deep reddish-brown, stipitate or rarely subsessile apothecia, a gelatinized ectal excipulum of textura porrecta covered by ochre-brown cortical hyphae with short outgrowths, inamyloid asci arising from simple septa, and large, multiguttulate, ellipsoid ascospores. Especially the latter varied among the collections, particularly in width, some being predominantly narrowly ellipsoid, the others more broadly ellipsoid. The living paraphyses usually looked empty and colourless by lacking vacuolar bodies (VBs), but sometimes they contained groups of lipid bodies (LBs). The pale to bright ochre-brown cortical hyphae of the receptacle and stipe often had an encrusted surface but were occasionally smooth.

In order to summarize the most important differences between *Bryorutstroemia* and related genera, the following key is provided. It needs to be taken as provisional, as the taxonomy of *Rutstroemiaceae* is still insufficiently solved and nomenclatural changes in the circumscription of the family and its members can be expected.

 

**Provisional key to the recognized genera of *Rutstroemiaceae* s.l.**

1. Asci (†) with prominent, inamyloid apical wall thickening; ascospores permanently hyaline; growing on bryophytes........ ***Bryorutstroemia***
1. Ascus apex (†) with amyloid apical ring of the *Sclerotinia*-type, rarely faintly amyloid or inamyloid, but then only moderately thick-walled; growing on phanerogams........ 2
2. On monocotyledons........ ***Clarireedia***
2. On dicotyledons or gymnosperms........ 3
3. Apothecia externally with prominent, septate, thick-walled setae........ ***Torrendiella***
3. Apothecia without setae........ 4
4. Ascospores permanently hyaline........ ***Rutstroemia*** (including *Dencoeliopsis*), ***Lanzia***
4. Ascospores turning brown with age, either within the living asci or when overmature........ ***Bicornispora***, ***Lambertella***, ***Martininia***

### 4.2. Phylogenetic Remarks

Based solely on cultural isolates, Salgado-Salazar et al. described three new species in the new genus *Clarireedia* in 2018 [21] and Hu et al. added a fourth species, *C. paspali* Jian Hu and Lamour in 2019 [24]. Because teleomorphs were absent in their samples, the authors overlooked close relationships of their *Clarireedia* spp. with old taxa recognized in *Rutstroemia*. For instance, their wide concept of *Clarireedia bennettii* C. Salgado et al. encompasses ITS sequences which fully match GenBank uploads under the names *R. calopus* (Fr.) Rehm, *R. henningsiana* (Plöttn.) Dennis, and *R. paludosa* (E.K. Cash and R.W. Davidson) J.W. Groves and M.E. Elliott, here classified as *Clarireedia calopus* and *C. henningsiana* (Figure 9 and Figure 10).

The type clade of *Clarireedia homoeocarpa* is closely related to *R. maritima* (Roberge ex Desm.) Dennis and *R. asphodeli* (Duvernoy and Maire) R. Galán and Matočec, here classified as *Clarireedia maritima* and *C. asphodeli*, whereas the remaining three species (*Clarireedia jacksonii* C. Salgado et al., *C. monteithiana* C. Salgado et al., *C. paspali*) represent a distinct group of genotypes which includes strains that are misnamed as *Sclerotinia homoeocarpa* in GenBank (based on our ML analysis of ITS rDNA, not shown).

Delimitation of the families *Sclerotiniaceae* and *Rutstroemiaceae* within the sclerotiniaceous lineage is still not clear in all respects. In the morphology-based classification defined by ascospores with a low vs. high lipid content coupled with globose vs. prismatic excipular cells, respectively, both families are paraphyletic (Figure 9 and Figure 10). Hereafter, *Scleromitrula*, *Martininia*, and *Piceomphale* share characters with the core clade of *Sclerotiniaceae*, while *Lambertella* and *Clarireedia* share characters with the core clade of *Rutstroemiaceae*. Additionally, *Bryorutstroemia* shares characters with *Rutstroemiaceae*, for which it could represent an ancestor on a phylogenetically old host, although the tree topology of Figure 9 suggests an evolution from mainly woody plants to monocots and mosses. The current concept that characterizes *Rutstroemiaceae* by a stroma and *Sclerotiniaceae* by sclerotia [25] largely coincides with the morphology-based concept, but both concepts include some problematic genera.

The difficulty of conducting phylogenetic analysis on sclerotiniaceous fungi based on rDNA data alone became obvious when trying to resolve the position of *Schroeteria* [23]. Multigene analyses probably better resolve phylogenetic affinities in this group. However, in a preliminary analysis of the *EF1α* gene with MEGA6 (TN+G, not shown), which comprised members of *Helotiales* (mainly sclerotiniaceous taxa), *Pezizales*, *Phacidiales*, *Rhytismatales*, *Dothideomycetes*, *Eurotiomycetes*, and *Sordariomycetes*, *B. fulva* clustered with *Sordariomycetes*, though with a high distance. *B. fulva* formed a clade with *Clarireedia* only when non-helotialean sequences were excluded from the analysis (Figure S3). Despite this curious result, BLAST search (megablast) for *EF1α* (strain Z.S. 19/2021) yielded *Rutstroemia firma* as the second most similar species (85.3%, query cover 61%), with the highest similarity of 88% (query cover 51%) to *Spathularia* (*Rhytismatales*) and 83.5–83.6% (query cover 68%) to *Sordaria* (*Sordariomycetes*) and *Lasiobolidium* (*Pezizales*). BLASTn search, however, yielded *R. firma* on top with 84.4% similarity (80% query cover). The *EF1α* sequences obtained from *B. fulva* strongly deviate at various positions from any other group of *Ascomycota*, which impedes a reasonable conclusion about its phylogenetic relationships. *EF1α* sequences obtained from four collections of *B. fulva* in this study were about the same length of 500 nucleotides and fully identical (except for nine ambiguities in C.N. 103), thus confirming the reliability of the result.

### 4.3. Ecological Remarks

*Bryorutstroemia fulva* is a necrotrophic parasite, causing bleaching of the host tissues. These striking substrate discolourations help one to spot the apothecia in the field (Figure 3), similarly as in several other species of bryophilous *Helotiales*, such as *Belonioscyphella hypnorum* [26], *Bryoscyphus dicrani* (pers. obs.), *B*. *hyalotectus* [27], and *Roseodiscus subcarneus* [28]. In the present study, *B. fulva* has been collected on mosses of the family *Dicranaceae* (*Dicranales*), mostly *D. heteromalla*. Only a single collection from Sweden grew on a moss from a different family and order, *Bucklandiella heterosticha* (≡ *Racomitrium heterostichum*, *Grimmiaceae*, *Grimmiales*). Suitable localities are shaded surfaces of acidic bedrock, very often in planted spruce forests. In several collections *B. fulva* grew more or less remotely associated with *Mniaecia jungermanniae*, a common hepaticolous ascomycete with deep blue apothecia (observed in collections J.P.P. 24120, Z.S. 23/2021, 44/2021, 48/2021), and *M.* cf. *gemmata* with whitish apothecia (J.P.P. 15051, Z.S. 18/2021, 23/2021).

We have encountered *B. fulva* mainly during the colder season, i.e., from November to May, but three of our records were from July. Collections from August by J. Poelt [29], September by A. Yelland [17], and October by D.M. Henderson [29] also exist. Although only a few records have been published, *B. fulva* seems to be common in colline to montane regions with acidic bedrock, which was exemplified in the present study for Czechia (24 collections during 2021–2022). The presently known distribution (Figure 11) is certainly incomplete. However, as the most frequent host *D. heteromalla* prefers acidic pH and grows most often on acidic forest soil or less often on sandy soil or directly on silicate boulders [30], the fungus might be rarer in areas with neutral to basic soil.

In France (Bretagne), the Netherlands, Luxembourg, Germany, Poland (Silesia), and Czechia the host was always *Dicranella*, which mainly grew on acidic sandstone (Figure 2: 1a), but also on sandy or loamy soil over sandstone, slate (Ordovician shale), silt, orthogneiss, migmatite, or granulite, etc. Sometimes the moss grew on soil on an uprooted fallen tree. The vegetation was preferably an acidic pure coniferous forest (predominantly *Picea* but also *Pinus*), also mixed with *Betula* or *Fagus,* etc. In Divadlo, the main vegetation was a *Vaccinio myrtilli-Pinetum sylvestris*, in Stohánek (Figure 3: 1) a *Vaccinio vitis-idaeae-Quercetum* with *Pinus sylvestris* and *Quercus petraea*, less often *Q. robur*, with admixture of *Betula pendula*, *Sorbus aucuparia*, and *Frangula alnus*, but also *Dicrano-Pinion* with the dominant *P*. *sylvestris* admixed with *Quercus petraea*, *Betula pendula*, *Frangula alnus* or *Sorbus aria*. Collections were often from the margins of forest pathways and also in ditches at the edges of roads. At the French sites the host moss occurred in close association with *Diplophyllum albicans*, *Calypogeia*, and *Cephalozia,* etc. Especially when growing on rock, the plant community in which *Bryorutstroemia fulva* parasitizes *Dicranella* may be classified as *Dicranellion heteromallae* [31]. In Sweden *B. fulva* grew on *Bucklandiella* (Figure 8: 2a) or *Dicranum* covering silicate stonewalls, and at the Hungarian site it grew in cushions of *D. scoparium* occurring scattered on open soil in an acidophilous *Quercus petraea* forest (Figure 8: 1a).

**Figure 1:**
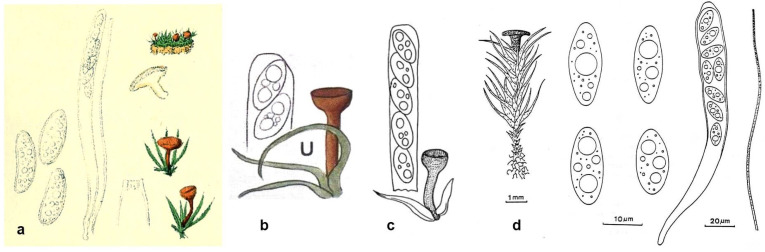
*Bryorutstroemia fulva* as illustrated under the name *Helotium fulvum* by (**a**) Boudier (1897 [1] pl. III fig. III), (**b**) Dennis (1978 [2] pl. XVIII U), (**c**) Ellis et Ellis (1988 [32] fig. 5), and (**d**) De Meulder (1992 [3] p. 80).

**Figure 2:**
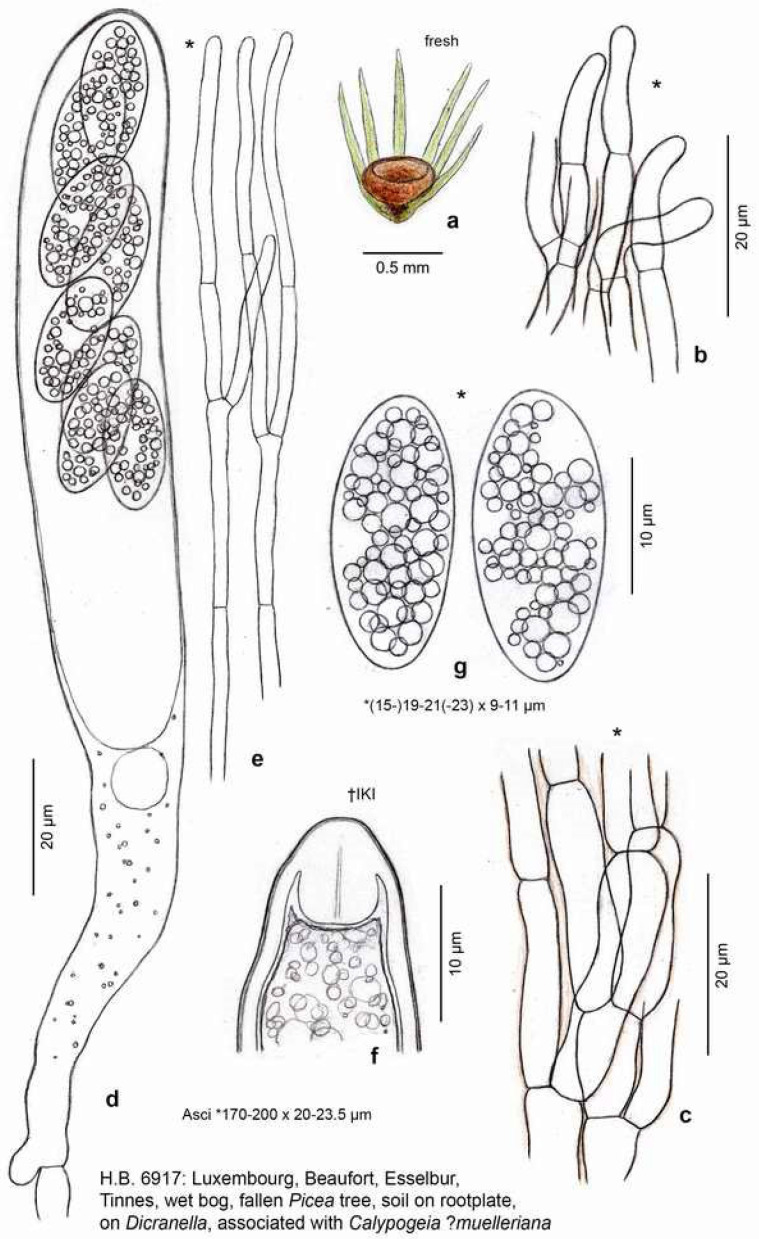
*Bryorutstroemia fulva* on *Dicranella* cf. *heteromalla*. (**a**) fresh apothecium formed in leaf axils at tip of plant; (**b**) hair-like marginal elements; (**c**) ectal excipular cells in surface view; (**d**) mature ascus; (**e**) upper part of paraphyses; (**f**) apex of immature ascus with prominent wall thickening expanding into the ascoplasm; (**g**) mature ascospores containing numerous LBs. Living state, except for f (in IKI).—Del. H.O. Baral.

**Figure 3:**
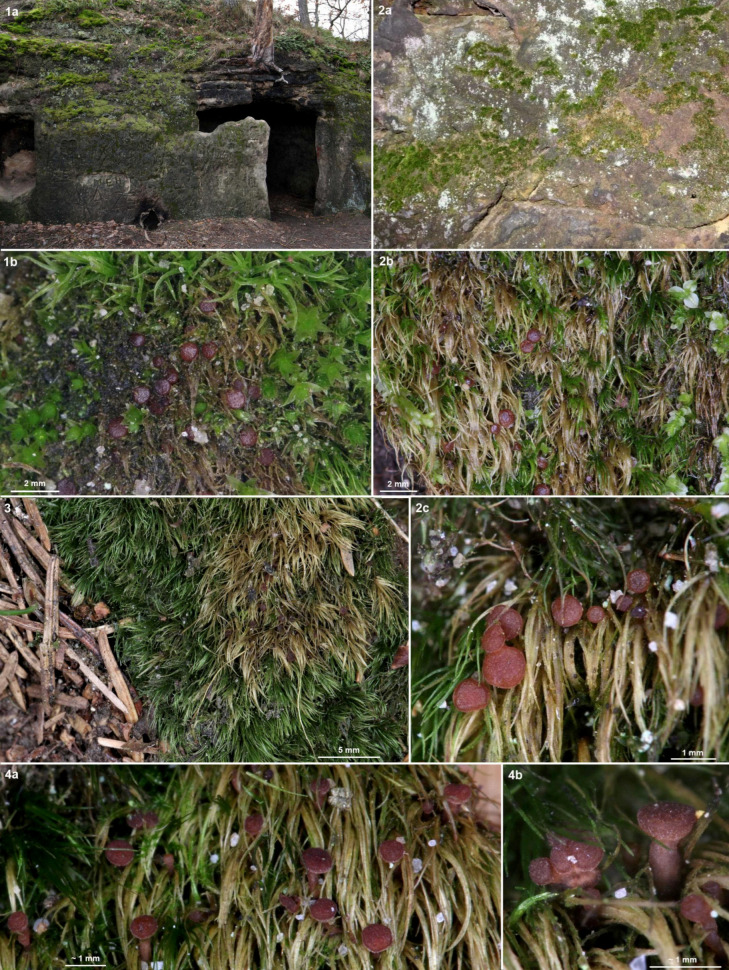
*Bryorutstroemia fulva* on *Dicranella* growing on sandstone in northern Bohemia. (**1a**,**2a**) collection sites; (**1b,2b**,**c**,**3,4a**,**b**) apothecia in leaf axils of bleached leaves.—(**1**) Stohánek (Z.S. 9/2021), (**2**) Sloup v Čechách (Z.S. 11/2021), (**3**) Petrovice (Z.S. 17/2021), (**4**) Sloup v Čechách (Z.S. 2/2021). Phot. Z. Sochorová.

**Figure 4:**
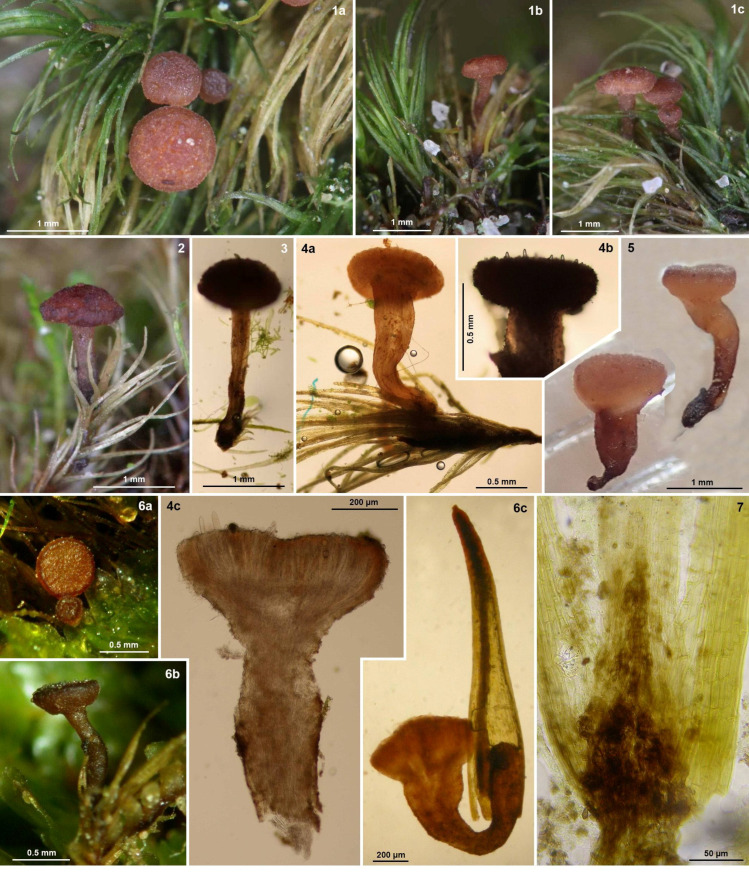
*Bryorutstroemia fulva* in leaf axils of *Dicranella* (from northern Bohemia, Bretagne, and Luxembourg) or *Dicranum* (from Hungary). (**1a**–**c**,**2**,**5**,**6a**,**b**) apothecia in reflected light; (**3**,**4a**,**b**,**6c**) apothecia in transmitted light (**4b** showing protruding mature asci); (**4c**) median section of apothecium; (**7**) lower part of leaf with brown fungal tissue. (**1**–**5**,**6a**,**b**) fresh apothecia, (**6c**) rehydrated apothecium; (**4a**–**c**,**6c**) in water, (**7**) in PVA.—(**1**–**4**) phot. Z. Sochorová: (**1**) Sloup v Čechách (Z.S. 11/2021), (**2**) Janovice v Podještědí (Z.S. 16/2021), (**3**) Jablonné v Podještědí (Z.S. 15/2021), (**4**) Sloup v Čechách (Z.S. 2/2021); (**5**) phot. C. Németh: Budakeszi (C.N. 103), (**6a**,**b**) phot. J.P. Priou, (**6c**) H.O. Baral: Sixt-sur-Aff (J.P.P. 26054, H.B. 8083), (**7**) phot. H.O. Baral: Beaufort (H.B. 6917).

**Figure 5:**
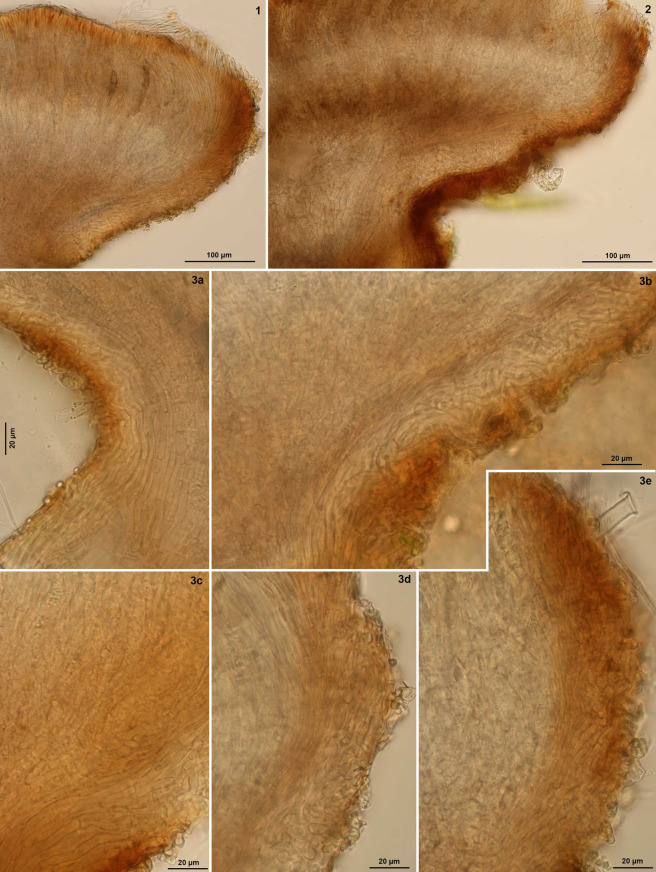
*Bryorutstroemia fulva* on *Dicranella* (from northern Bohemia). Median section of apothecia: (**1**,**2**) receptacle, (**3a**,**b**) upper stipe and lower flanks, (**3c**) lower flanks, (**3d**,**e**) margin. Living state.—(**1**) Divadlo (Z.S. 8/2021); (**2**) Besedice (Z.S. 7/2021); (**3**) Sloup v Čechách (Z.S. 2/2021). Phot. Z. Sochorová.

**Figure 6:**
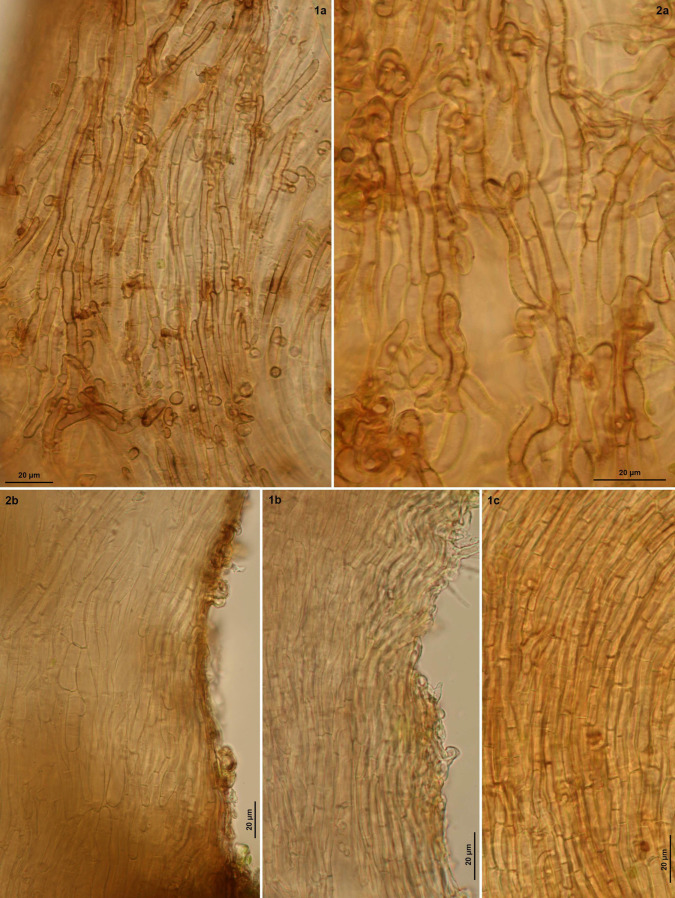
*Bryorutstroemia fulva* on *Dicranella* (from northern Bohemia). Apothecial stipe in surface view (**1a**,**c**,**2a**) and median section (**1b**,**2b**). Living state.—(**1**) Sloup v Čechách (Z.S. 2/2021); (**2**) Divadlo (Z.S. 8/2021). Phot. Z. Sochorová.

**Figure 7:**
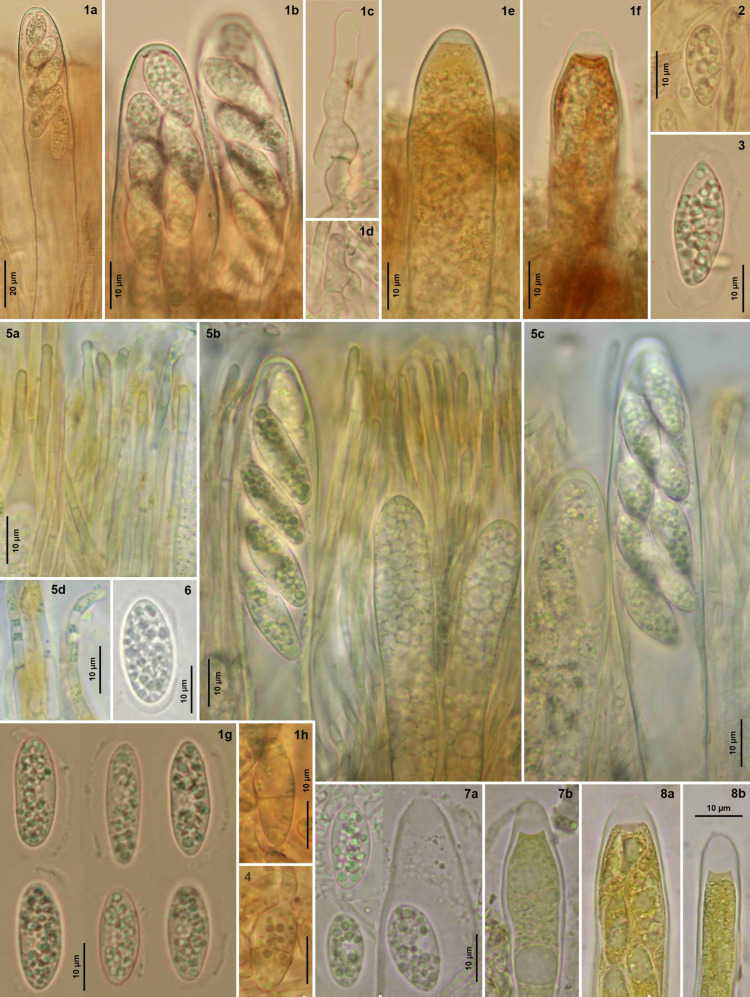
*Bryorutstroemia fulva*. (**1a**,**b**,**5b**,**c**,**7a**,**8a**) mature asci; (**1c**,**d**,**5c left**,**7b**,**8b**) immature asci; (**5b right**) young asci; (**5a**,**b**,**d**) paraphyses; (**1e**,**2**,**3**,**6**,**7a**) mature ascospores; (**1f**,**4**) overmature ascospores. Living state, except for (**7a**) asci, in H_2_O, (**7b**,**8**) in IKI.—(**1**–**4**) from northern Bohemia, on *Dicranella* (phot. Z. Sochorová), (**1**) Z.S. 2/2021, (**2**) Z.S. 15/2021, (**3**) Z.S. 16/2021, (**4**) Z.S. 17/2021; (**5**) Hungary, on *Dicranum* (phot. C. Németh, C.N. 103); (**6**) Sweden, on *Bucklandiella* (phot. R. Isaksson, UPS F-990878); (**7**,**8**) France, on *Dicranella*, (**7**) phot. J.P. Priou, J.P.P. 15051, (**8**) H.O. Baral, H.B. 8083.

**Figure 8:**
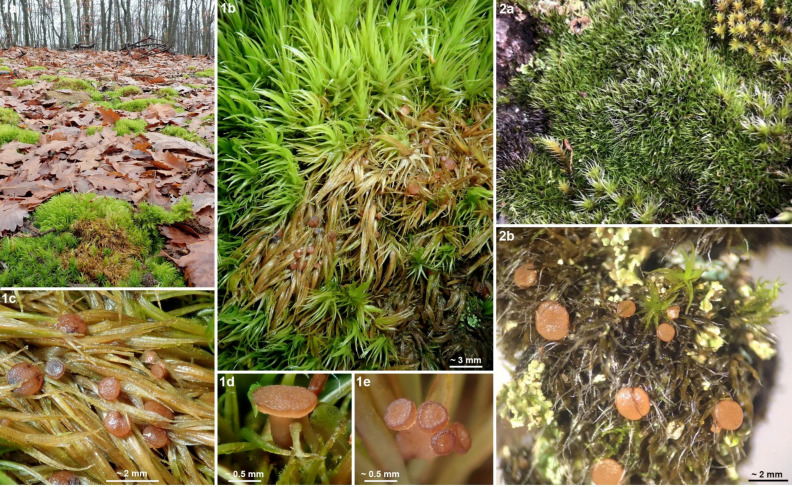
*Bryorutstroemia fulva*. (**1a**) collection site in *Quercus petraea* forest, (**1b–e**) apothecia in cushions of *Dicranum scoparium* (Hungary, phot. C. Németh, C.N. 103); (**2a**,**b**) *Bucklandiella hetero-sticha* on silicate stonewall (Sweden, phot. R. Isaksson, UPS F-990878).

**Figure 9:**
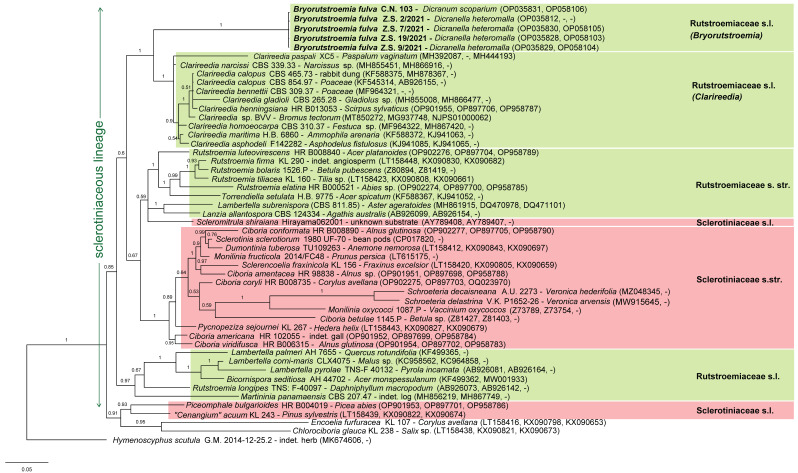
Bayesian analysis of the sclerotiniaceous lineage which comprises *Rutstroemiaceae* s.l. and *Sclerotiniaceae* s.l., based on ITS1-5.8S-ITS2 and LSU D1–D3 rDNA and *EF1α*. The chosen outgroup comprises members of *Cenangiaceae*, *Chlorociboriaceae*, and *Helotiaceae*.

**Figure 10:**
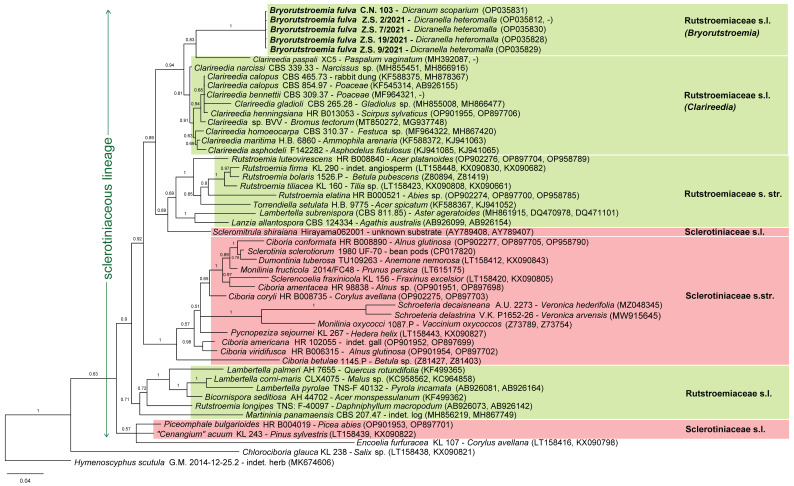
Bayesian analysis of the sclerotiniaceous lineage based on ITS1-5.8S-ITS2 and LSU D1–D3 rDNA.

**Figure 11:**
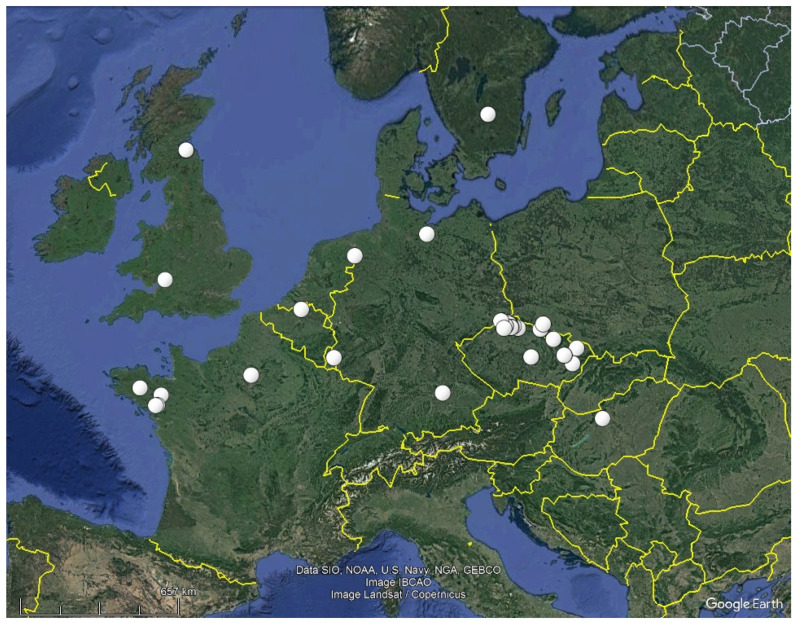
Known distribution of *Bryorutstroemia fulva* in Europe (white dots referring to the collections cited under “Specimens included”).

### 4.4. Literature Reports

Boudier [1] described the apothecia of *H. fulvum* with a diameter and height of 0.5–1.5 mm, asci 150–200 × 17–18 µm, paraphyses apically slightly widened to 3–4 µm, eguttulate, and ascospores oblong-ellipsoid, subinaequilateral, rarely somewhat curved, *16–21 × 7–10 µm, multiguttulate (see Figure 1a). He illustrated bright reddish-brown apothecia but described them as brown to yellow-brown (“brunneo-fulvum”) or fawn-brownish (“fauve brunâtre”), with hymenium and stipe base the most deeply coloured. He apparently did not test the asci with iodine and did not observe overmature spores as he stated that the spores were never septate. When taking the ascus width in Boudier’s drawing as 17 µm, ascus length becomes 250 µm, spores in the asci 18–21 × 7–7.5 µm, and paraphysis width about 4 µm. Evaluation of the scales based on the 225× and 820× magnifications yield values of *295 × 18.5 µm for the ascus, *21–22 × 7–8.5 µm for the free ascospores, and 4 µm for the paraphysis, suggesting some scale and length/width error in Boudier’s drawing regarding ascus length (Figure 1a).

British records of *H. fulvum* from leaf axils of *Dicranella heteromalla* were figured by Dennis [2] (as ‘*D. heteromera*’) and Ellis et Ellis [32], but no collection data were given (see Figure 1b,c). The database of the British Mycological Society [17] indicates two collections, one from Gloucestershire made in 1991 and one without data. Dennis mentioned the negative ascus iodine reaction and considered an affinity with *Rutstroemia*, but also referred to a “small group of similar species parasitic on bryophytes, for which a separate genus may perhaps be needed” (Dennis probably meant the later erected genus *Bryoscyphus* Spooner). The almost identical measurements by Dennis and Ellis et Ellis (l.c., asci 150–180 × 13–16 µm, ascospores 16–21 × 6–9 µm) concur well with the present data. The ascospores were illustrated with two large and some smaller LBs, probably because the material was studied in a rehydrated state.

Much earlier, Dennis ([33] p. 58) compared *H. fulvum*, based on Boudier’s description, with a collection on *Lycopodium* from Norway which he identified as *Poculopsis ogrensis* Kirschst. This species he combined as *Allophylaria ogrensis* (Kirschst.) Dennis, although the inner ectal excipulum was drawn with thin-walled cells and the texture described as very soft. Contrary to *H. fulvum*, the apothecia were yellow when fresh but turned dark brown on drying, and the much shorter asci had an amyloid ring. At that time, Dennis did not know *H. fulvum* by personal study. For *A. ogrensis,* he saw some similarities with the *Sclerotiniaceae* (as *Ciborioideae*), but the absence of a substratal blackening or a sclerotium excluded such a relationship.

De Meulder [3] described and illustrated a personal collection of *H. fulvum* on *Dicranella cerviculata* collected in 1992 in Belgium (Figure 1d). Ten days after this collection was made, the first author received a part of the dried specimen from the collector. Despite the short time span, no living elements could be found. The obtained measurements differed from De Meulder’s data by much narrower paraphyses (†2–2.2 vs. †3–4 µm), slightly shorter asci (†130–158 × 14–18 vs. †137.5–175 × 12.5–18 µm), and distinctly narrower ascospores (†15–20 × 6–7.5 vs. *15.5–22.75 × 7.5–8.7 µm). De Meulder might have studied a rehydrated apothecium with still living spores, judging from the larger size, and also from the included partly large LBs which were likely formed by the fusion of smaller ones during rehydration. Paraphysis width is hardly over 1.5 µm when evaluated from De Meulder’s drawing, hence his given width of 3–4 µm should be an error or a mere copy of Boudier’s data, whereas values around †1.5–2.5 µm would have been closer to what was here observed in the other collections.

### 4.5. Misinterpretations

Under the name *Helotium fulvum*, Velenovský ([34] p. 209) gave an unillustrated record on *Hylocomium splendens*, *H. squarrosum* (≡ *Rhytidiadelphus squarrosus*), and *Hypnum cupressiforme*. When revising the cited collection, Svrček ([35] p. 149, pl. 19 fig. 7) found only *Hylocomium splendens* inside the voucher, with apothecia on the leaves, and concluded that it is a species very different from *H. fulvum*, for which he could not give a name. The ascospores were much more slender (†17–19 × 4–4.5 µm), with two large guttules, and the inamyloid asci much smaller (†90–100 × 6–10 µm, Velenovský: †100–120 × 5–8 µm) compared to Boudier’s *H. fulvum*, with a strongly inflated foot (crozier?). The apothecia were 1–1.5 mm diam., blackish-brown, sessile or short-stalked. Svrček’s description suggests a species of *Hymenoscyphus* s.l. Another specimen found in Velenovský’s herbarium under the name *H. fulvum* was on *Rhytidiadelphus squarrosus*, and Svrček (l.c.) identified it as *Hymenoscyphus rhytidiadelphi* (≡ *Bryoscyphus rhytidiadelphi*).

*Bryorutstroemia fulva* may be confused with *Bryoscyphus dicrani* because of a similar ascospore size and shape and inamyloid asci. However, confusion is only possible when comparing herbarium specimens in which *B. dicrani* may attain a reddish-brownish colour due to secondary pigmentation of the multiguttulate contents of paraphyses and excipular cells. In the living state, *B. dicrani* has white apothecia, binucleate ascospores with a lower lipid content (OCI 2–3), and multiguttulate paraphyses and cortical excipular cells due to strongly refractive vacuolar bodies (VBs). A further difference lies in the asci which are also inamyloid but arise from croziers.

Hengstmengel [19] studied a collection on *Brachythecium rutabulum* from the Netherlands (Drenthe, Rolde, Deurzerbroek). We have seen no documentation of this collection, but we consider the possibility that it might be a misidentification, judging from the deviating host.

### 4.6. Other Bryicolous Species of the Sclerotiniaceous Lineage

*Bryorutstroemia fulva* is exceptional within the sclerotiniaceous lineage by its ecological restriction to acrocarpous mosses. Only a very small number of other bryicolous discomycetes with a clear affinity to the sclerotiniaceous lineage are known up to now. One of them is *Sclerotinia atrostipitata* Svrček from Czechia, which was described as emerging from a 2 mm large subglobose sclerotium among rhizoids of *Ceratodon purpureus*, with globose excipular cells, amyloid asci, and comparatively small, ellipsoid-ovoid, eguttulate ascospores [36]. Svrček’s remark of an attachment of the sclerotium to the moss rhizoids might be an argument for a real connection to the moss, but interactions at the cellular level have not been assessed. The North American *Sclerotinia incondita* (Ellis) Sacc. mentioned by Svrček likewise grew among mosses, but its description which includes four-spored asci is too brief to permit any conclusion.

## Figures and Tables

**Table 1:** Sequences included in phylogenetic analysis (T = type). Newly generated sequences in bold. - = gene region missing, ? = data missing, ^#^ = as *Rutstroemia cuniculi*.

Species	Collection Number	Country	Host	ITS	LSU	*EF1α*
*Bicornispora seditiosa*	AH 44702 T	Spain	*Acer monspessulanum*	KF499362	KF499362	MW001933
***Bryorutstroemia fulva***	**C.N. 103**	**Hungary**	***Dicranum scoparium***	**OP035831**	**OP035831**	**OP058106**
***Bryorutstroemia fulva***	**Z.S. 2/2021**	**Czech Republic**	***Dicranella heteromalla***	**OP035812**	**-**	**-**
***Bryorutstroemia fulva***	**Z.S. 7/2021**	**Czech Republic**	***Dicranella heteromalla***	**OP035830**	**OP035830**	**OP058105**
***Bryorutstroemia fulva***	**Z.S. 9/2021**	**Czech Republic**	***Dicranella heteromalla***	**OP035829**	**OP035829**	**OP058104**
***Bryorutstroemia fulva***	**Z.S. 19/2021**	**Czech Republic**	***Dicranella heteromalla***	**OP035828**	**OP035828**	**OP058103**
*“Cenangium” acuum*	KL 243	Germany	*Pinus sylvestris*	LT158439	KX090822	KX090674
*Chlorociboria glauca*	KL 238	France	*Salix* sp.	LT158438	KX090821	KX090673
***Ciboria amentacea***	**HR 98838**	**Czech Republic**	***Alnus*** **sp.**	**OP901951**	**OP897698**	**OP958788**
***Ciboria americana***	**HR 102055**	**Czech Republic**	**indet. gall**	**OP901952**	**OP897699**	**OP958784**
*Ciboria betulae*	1145.P	Norway	*Betula* sp.	Z81427	Z81403	-
***Ciboria conformata***	**HR B008890**	**Czech Republic**	***Alnus glutinosa***	**OP902277**	**OP897705**	**OP958790**
***Ciboria coryli***	**HR B008735**	**Czech Republic**	***Corylus avellana***	**OP902275**	**OP897703**	**OQ023970**
***Ciboria viridifusca***	**HR B006315**	**Czech Republic**	***Alnus glutinosa***	**OP901954**	**OP897702**	**OP958783**
*Clarireedia* *asphodeli*	F142282	Spain	*Asphodelus fistulosus*	KJ941085	KJ941065	-
*Clarireedia bennettii*	CBS 309.37	unknown	indet. *Poaceae*	MF964321	-	-
*Clarireedia* *calopus*	CBS 854.97	Netherlands	indet. *Poaceae*	KF545314	AB926155	-
*Clarireedia calopus* ^#^	CBS 465.73	Great Britain	rabbit dung	KF588375	MH878367	-
*Clarireedia* *gladioli*	CBS 265.28 T	unkown	*Gladiolus* sp.	MH855008	MH866477	-
***Clarireedia henningsiana***	**HR B013053**	**Czech Republic**	***Scirpus sylvaticus***	**OP901955**	**OP897706**	**OP958787**
*Clarireedia homoeocarpa*	CBS 310.37	Great Britain	*Festuca* sp.	MF964322	MH867420	-
*Clarireedia maritima*	H.B. 6860	Spain	*Ammophila arenaria*	KF588372	KJ941063	-
*Clarireedia* *narcissi*	CBS 339.33	Netherlands	*Narcissus* sp.	MH855451	MH866916	-
*Clarireedia paspali*	XC5	China	*Paspalum vaginatum*	MH392087	-	MH444193
*Clarireedia* sp.	BVV	USA	*Bromus tectorum*	MT850272	MG937748	NJPS01000062
*Dumontinia tuberosa*	TU109263	Estonia	*Anemone nemorosa*	LT158412	KX090843	KX090697
*Encoelia furfuracea*	KL 107	Estonia	*Corylus avellana*	LT158416	KX090798	KX090653
*Hymenoscyphus scutula*	G.M. 2014-12-25.2	Luxembourg	indet. herb	MK674606	MK674606	-
*Lambertella corni-maris*	CLX4075	USA	*Malus* sp.	KC958562	KC964858	-
*Lambertella palmeri*	AH 7655	Spain	*Quercus rotundifolia*	KF499365	KF499365	-
*Lambertella pyrolae*	TNS-F 40132 T	Japan	*Pyrola incarnata*	AB926081	AB926164	-
*Lambertella subrenispora*	CBS 811.85	Japan	*Aster ageratoides*	MH861915	DQ470978	DQ471101
*Lanzia allantospora*	CBS 124334	New Zealand	*Agathis australis*	AB926099	AB926154	-
*Martininia panamaensis*	CBS 207.47	Panama	indet. log	MH856219	MH867749	-
*Monilinia fructicola*	2014/FC48	Hungary	*Prunus persica*	LT615175	LT615175	-
*Monilinia oxycocci*	1087.P	Norway	*Vaccinium oxycoccos*	Z73789	Z73754	-
***Piceomphale bulgarioides***	**HR B004019**	**Czech Republic**	***Picea abies***	**OP901953**	**OP897701**	**OP958786**
*Pycnopeziza sejournei*	KL 267	France	*Hedera helix*	LT158443	KX090827	KX090679
*Rutstroemia bolaris*	1526.P	Norway	*Betula pubescens*	Z80894	Z81419	-
***Rutstroemia elatina***	**HR B000521**	**Czech Republic**	***Abies*** **sp.**	**OP902274**	**OP897700**	**OP958785**
*Rutstroemia firma*	KL 290	Estonia	indet. angiosperm	LT158448	KX090830	KX090682
*Rutstroemia longipes*	TNS: F-40097	Japan	*Daphniphyllum macropodum*	AB926073	AB926142	-
***Rutstroemia luteovirescens***	**HR B008840**	**Czech Republic**	***Acer platanoides***	**OP902276**	**OP897704**	**OP958789**
*Rutstroemia tiliacea*	KL 160	Germany	*Tilia* sp.	LT158423	KX090808	KX090661
*Schroeteria decaisneana*	A.U. 2273	Germany	*Veronica hederifolia*	MZ048345	MZ048345	-
*Schroeteria delastrina*	V.K. P1652-26	Germany	*Veronica arvensis*	MW915645	MW915645	-
*Sclerencoelia fraxinicola*	KL 156	Germany	*Fraxinus excelsior*	LT158420	KX090805	KX090659
*Scleromitrula shiraiana*	Hirayama062001	?	?	AY789408	AY789407	-
*Sclerotinia sclerotiorum*	1980 UF-70	USA	bean pods	CP017820	CP017820	-
*Torrendiella setulata*	H.B. 9775	Canada	*Acer spicatum*	KF588367	KJ941052	-

## Data Availability

Names of the new species and combinations were formally registered in MycoBank. Newly generated sequences were deposited in GenBank.

## Supplementary Materials

The following supplementary Bayesian analyses are available online at https://www.mdpi.com/article/10.3390/life13041041/s1; Figure S1: Bayesian analysis of ITS1-5.8S-ITS2 region; Figure S2: Bayesian analysis of D1–D2 domain of LSU rDNA; Figure S3: Bayesian analysis of *EF1α*; Figure S4: combined Maximum Likelihood analysis of dataset of Figure 9. For further data see legend to Figure 9 (as outgroup for S3 we selected *Chlorociboria glauca*).
