# Supplementary figures and images for: Bryorutstroemia (Rutstroemiaceae, Helotiales), a New Genus to Accommodate the Neglected Sclerotiniaceous Bryoparasitic Discomycete Helotium fulvum
Source: Life (Basel). 2023 Apr 18;13(4):1041. doi: 10.3390/life13041041 (PMC10144084; doi:10.3390/life13041041)

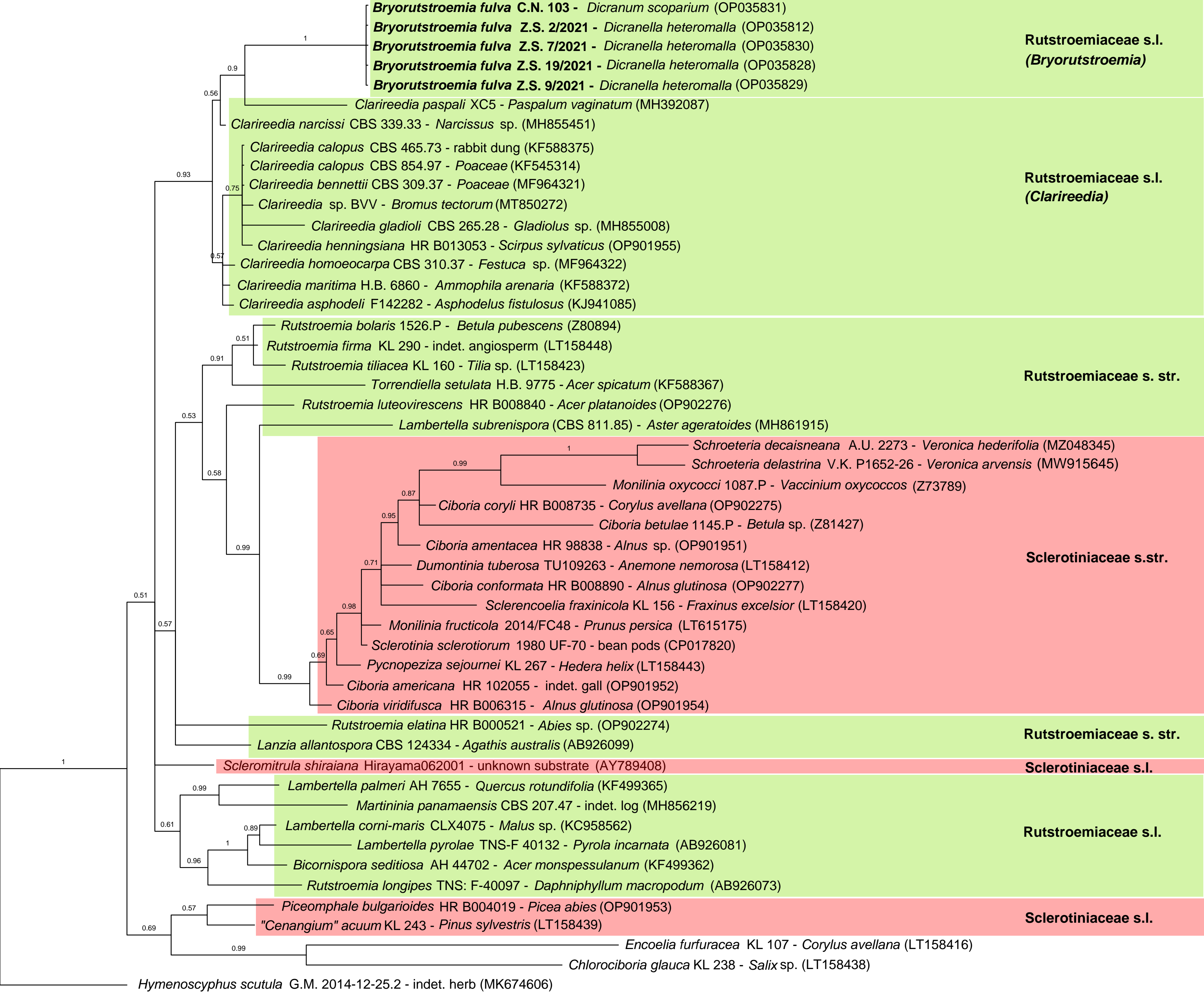

0.09

Supplement: Supplementary file 1 [file life-13-01041-s001.zip › Fig. S1 Bryorutstroemia ITS Bayes colours.pdf]

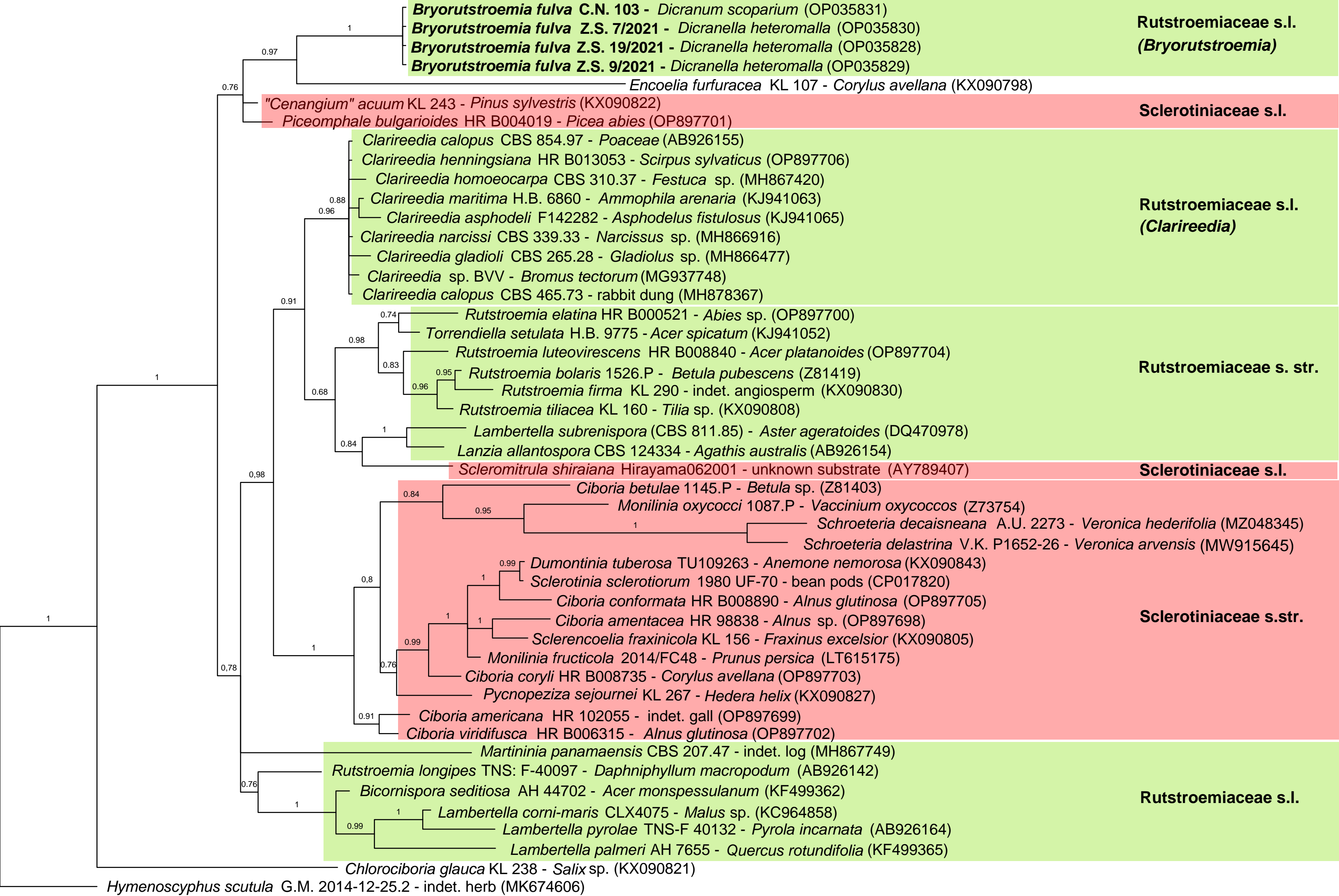

Supplement: Supplementary file 1 [file life-13-01041-s001.zip › Fig. S2 Bryoutstroemia LSU Bayes colours.pdf]

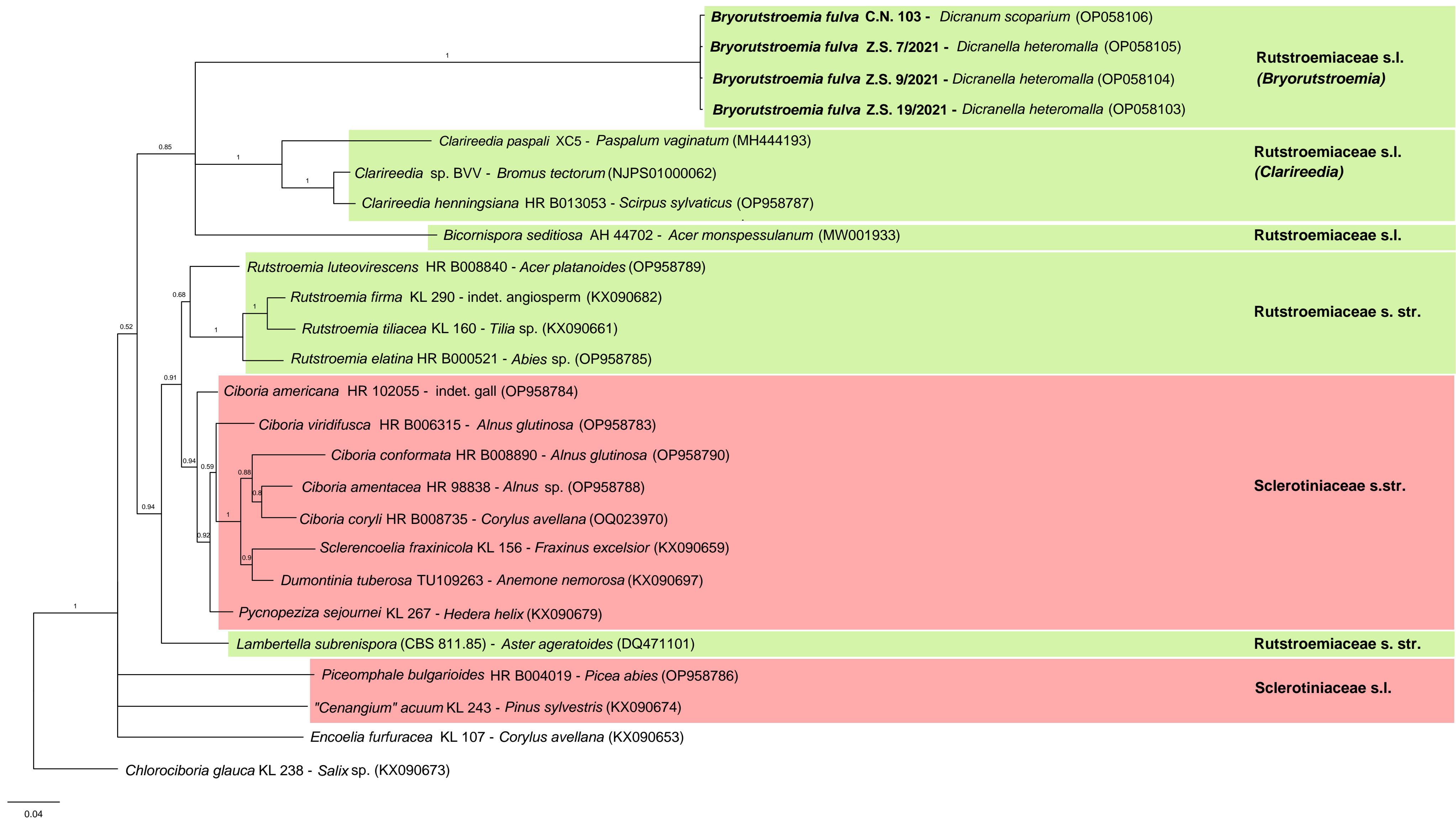

Supplement: Supplementary file 1 [file life-13-01041-s001.zip › Fig. S3 Bryorutstroemia TEF Bayes colours.pdf]

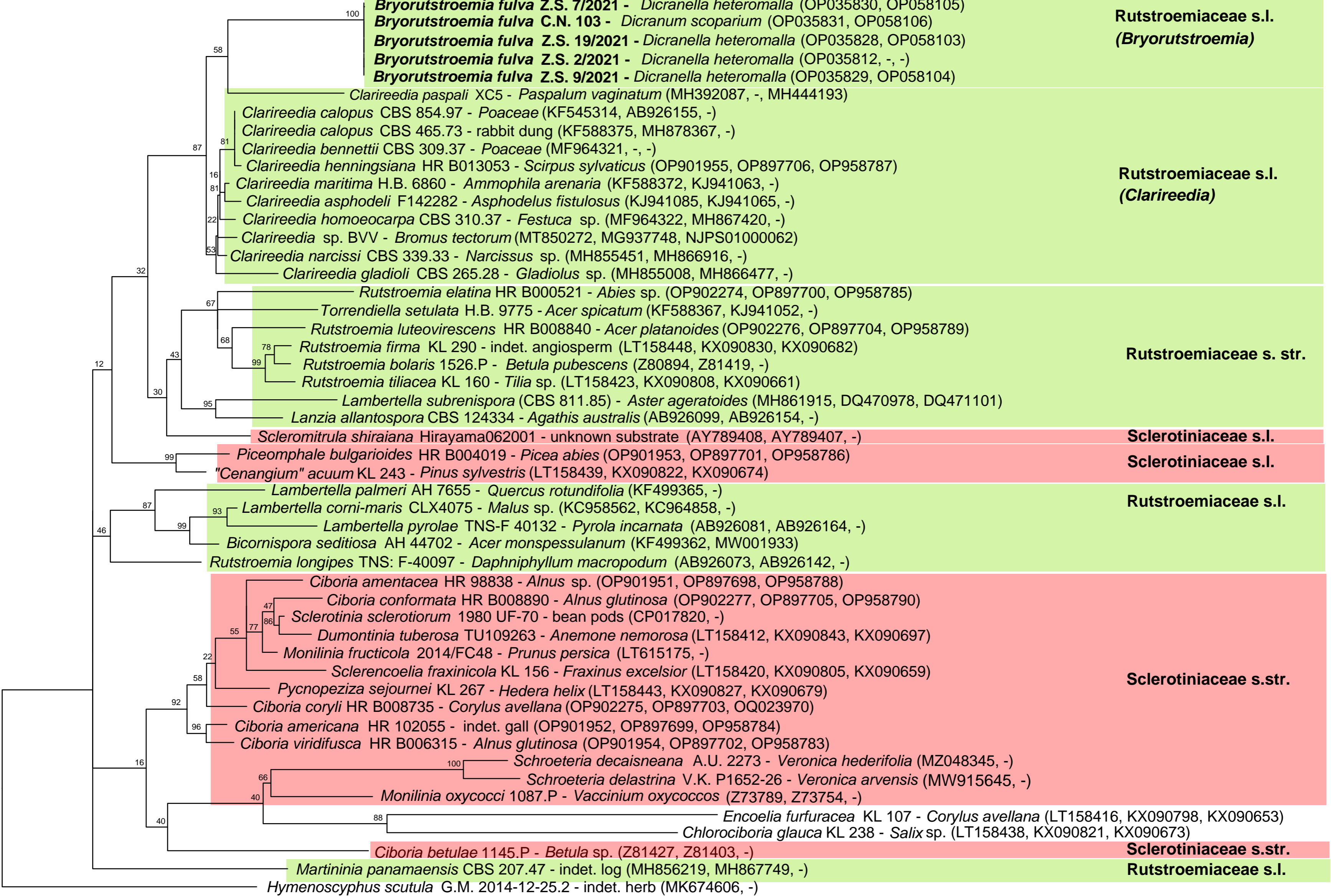

Supplement: Supplementary file 1 [file life-13-01041-s001.zip › Fig. S4 Bryorutstroemia ITS+LSU+TEF ML.pdf]
